# Non-Human Primates in Gabon: Occurrence Hotspots, Habitat Dynamics, Protected-Area Performance, and Conservation Challenges

**DOI:** 10.3390/biology15050405

**Published:** 2026-02-28

**Authors:** Mohamed Hassani Mohamed-Djawad, Barthelemy Ngoubangoye, Papa Ibnou Ndiaye, Krista Mapagha-Boundoukou, Neil Michel Longo-Pendy, Serge Ely Dibakou, Jean Nzue-Nguema, Désiré Otsaghe-Ekore, Stephan Ntie, Afred Ngomanda, Patrice Makouloutou-Nzassi, Mohamed Thani Ibouroi, Larson Boundenga

**Affiliations:** 1 Unité de Recherche en Ecologie de la Santé (URES), Centre Interdisciplinaire de Recherche Médicale de Franceville (CIRMF), Franceville BP 769, Gabon; mapaghakrista@gmail.com (K.M.-B.); longo2michel@gmail.com (N.M.L.-P.); patmak741@gmail.com (P.M.-N.); 2 Laboratoire de Biologie Évolutive Écologie et Gestion des Écosystèmes, Département de Biologie Animale, Faculté des Sciences et Techniques, Université Cheikh Anta Diop, Dakar BP 5005, Senegal; ibnou.ndiaye@ucad.edu.sn; 3 Groupe Thematique EcoConserve du CIRMF, Franceville BP 769, Gabon; genistha@hotmail.com (B.N.); sergeely@live.fr (S.E.D.); jean.nzue-nguema@etu.univ-lyon1.fr (J.N.-N.); otsaghe16@gmail.com (D.O.-E.); 4 Centre de Primatologie (CDP), Centre Interdisciplinaire de Recherche Médicale de Franceville (CIRMF), Franceville BP 769, Gabon; 5 Agence Nationale des Parcs Nationaux, Libreville BP 20379, Gabon; stephanntie@yahoo.fr; 6 Département de Biologie, Faculté des Sciences, Université des Sciences et Techniques de Masuku, Franceville BP 769, Gabon; 7 Département de Biologie et Ecologie Animale, Institut de Recherche en Ecologie Tropicale (IRET/CENAREST), Libreville BP 13354, Gabon; ngomanda@yahoo.fr; 8 Groupe de Recherches et de Protection de la Faune et de la Flore des Iles de l’océan Indien (GRPFOI), 6 rue Mnarajou, 97660 Dembeni, Mayotte, France; halibathani@yahoo.fr

**Keywords:** non-human primates, Gabon, land-cover change, protected areas, spatial hotspots, conservation planning

## Abstract

Gabon harbors one of Africa’s richest assemblages of non-human primates (NHPs), yet integrated national-scale evidence on their conservation status remains limited. To inform conservation strategies, we conducted the first nationwide assessment integrating habitat dynamics, the geographic distribution of species, and the effectiveness of the protected-area network in the country. We harmonized 300 m land-cover maps (ESA CCI 1992; Copernicus 2022), compiled 481 georeferenced occurrences, and identified concentration areas using kernel density estimation and Getis–Ord Gi* analysis. We quantified land-cover transitions with a per-pixel transition matrix and assessed protected-area capture using Monte Carlo randomization. Ten fully protected species are confirmed, including *Gorilla gorilla* and *Pan troglodytes*. Occurrences concentrate mainly in the Ogooué-Ivindo and Haut-Ogooué Provinces; ~10% of the national territory lies above the 90th kernel density percentile (≈26,700 km^2^), and 1.5% of cells qualify as hotspots at the 99% threshold. Primate records are strongly associated with evergreen broadleaved forests (87.9% of points), which remained persistent from 1992 to 2022 (forest-to-forest = 223,476 km^2^; 98.13%) with a net decline (−2571.66 km^2^; −1.19%). Gross losses (4046.58 km^2^) were mainly attributable to agricultural conversion (68.63%; χ^2^ = 31,525; *p* < 0.001). Over 90% of records fall in areas stable across 1992–2022. Protected areas (PAs) captured more occurrences (observed 40.1% vs. expected 18.47%; *p* < 0.001), yet gaps remain for some taxa (e.g., *Allochorocebus solatus*, 86% outside PAs). Overall, Gabon retains an extensive core of suitable habitat, but targeted action outside PAs and maintenance of landscape connectivity are needed to secure populations where agricultural expansion and fragmentation are intensifying.

## Simple Summary

Gabon is home to many species of monkeys and apes, including gorillas and chimpanzees, but conservation decisions often lack a clear national picture of where these animals are most frequently recorded, how their habitats have changed, and how well protected areas are covering them. We combined wildlife records collected during field missions and from public biodiversity databases with national land-cover maps from 1992 and 2022 to identify where primate observations are concentrated to measure how forests and other land types have changed over 30 years and to test whether protected areas contain more primate records than would be expected by chance. We found that primate records were strongly concentrated in the Ogooué-Ivindo and Haut-Ogooué regions and were mostly located in evergreen forests. This forest type remained largely intact between 1992 and 2022, but most forest loss that did occur was linked to conversion to agriculture. Protected areas contained more primate records than expected, showing that they contribute to conservation, but some species such as the sun-tailed monkey were still mostly recorded outside protected areas. These results can help guide land-use planning by protecting key forest connections, strengthening conservation actions outside protected areas, and reducing habitat loss where farming is expanding.

## 1. Introduction

The long-term survival of non-human primates (NHPs) is intricately linked to the quality, extent, and connectivity of their habitats [1], which are increasingly compromised by land-cover changes, resulting in habitat loss, fragmentation, and functional degradation [1,2]. These disturbances reduce food resource availability, elevate direct mortality risks (e.g., road kills and poaching), disrupt essential movement corridors, and heighten susceptibility to disease outbreaks and genetic isolation [3,4,5]. Currently, over 65% of primate species are classified as Vulnerable, Endangered, or Critically Endangered, with 93% facing population declines [1,2], underscoring an escalating conservation crisis primarily driven by intensified anthropogenic activities.

In Central Africa, tropical forests harbor some of the highest levels of primate biodiversity on the continent [6,7,8]. Gabon stands as a critical refuge within the Congo Basin, characterized by an exceptionally high forest retention, with approximately 84% of its territory covered by evergreen forest [9]. The country harbors a remarkable diversity of non-human primates, with at least 19 to 21 documented species [10]. Among these, 12 species are granted full legal protection, including iconic flagship taxa such as the western lowland gorilla (*Gorilla gorilla*), the central chimpanzee (*Pan troglodytes*), and the mandrill (*Mandrillus sphinx*) [10,11,12,13,14]. This positions the country as a crucial refuge within the Congo Basin and a strategic stronghold for conservation efforts [15,16,17].

Since the creation of 13 national parks in 2002, Gabon has established an ambitious institutional framework for conservation [18,19,20,21]. It is essential, however, to regularly evaluate the performance and capacity of this network, established over two decades ago, to withstand increasing anthropogenic pressures. Following the political transition of 31 August 2023 [22], and through the National Development Plan for the Transition (PNDT), the country has embarked on a large-scale development program simultaneously targeting road infrastructure, the rail network, the mining and forestry sectors, agricultural value chains, and hydraulic/energy works.

While this acceleration promises improved connectivity and economic growth, it can intensify habitat conversion and fragmentation; create barrier and edge effects along roads and railways; reduce landscape connectivity and sever corridors between protected areas; open new access fronts that facilitate poaching and illegal exploitation; increase human–wildlife conflict; and locally disrupt hydrological regimes, with repercussions for the quality and availability of resources.

In this context of rapid and expansive investment, it is crucial to have a robust, nationwide scientific evidence base to guide land-use planning, rigorously apply the mitigation hierarchy (avoid, minimize, restore, offset), and safeguard priority corridors before development.

Accordingly, this study presents the first integrated national assessment of primate conservation in Gabon, with a specific focus on the country’s 10 fully protected species. We specifically aim to: (i) map the distribution of occurrences and identify spatial clusters (hotspots/coldspots); (ii) quantify habitat dynamics between 1992 and 2022 and estimate the contribution of anthropogenic drivers (agriculture and urbanization); (iii) evaluate the spatio-temporal stability of habitat around occurrences and characterize local land-cover change trajectories; and (iv) evaluate the performance of the protected-area network (AP/OECM) by measuring the enrichment of NHP occurrences compared to random expectations (Monte Carlo randomization test).

## 2. Materials and Methods

### 2.1. Study Area

**Figure 1:**
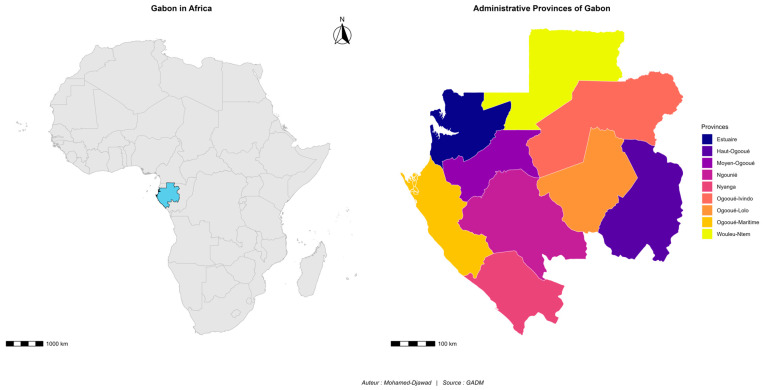
Geographical location of Gabon in Africa and its internal administrative divisions.

Gabon is a Central African country covering an area of 267,667 km^2^, with an estimated population of 2.3 million inhabitants in 2023. It is located between latitudes 2°15′ and 3°55′ south and longitudes 8°30′ and 14°30′ east [23]. Bordered to the west by the Atlantic Ocean, it shares land borders with Equatorial Guinea to the northwest, Cameroon to the north, and the Republic of the Congo to the east and south (Figure 1).

### 2.2. Land-Cover Data

Two global land-cover datasets were used to assess changes between 1992 and 2022. The 1992 land-cover layer was derived from the NetCDF product ESAC-CI-LC-L4-LCCS-Map-300m-P1Y-1992-v2.0.7, developed under the European Space Agency Climate Change Initiative (ESA CCI) [24]. The 2022 layer was obtained from C3S-LC-L4-LCCS-Map-300m-P1Y-2022-v2.1.1, produced by the Copernicus Climate Change Service (C3S) [25]. Both products belong to a consistent, cross-sensor land-cover time series and provide annual (P1Y), harmonized land-cover classifications at a 300 m spatial resolution based on the Food and Agriculture Organization of the United Nations Land Cover Classification System (LCCS) [26]. The analysis extent was delimited using the national administrative boundary shapefile (gadm36_GAB_0.shp) obtained from the GADM database (https://gadm.org/, accessed on 5 June 2025). All raster and vector processing was performed in R (v4.x) using the terra, sf, and dplyr packages, and all layers were handled in the WGS 84 coordinate system (EPSG:4326). The two land-cover rasters were co-registered prior to change detection: they were aligned to the same grid (identical extent, spatial resolution, origin, and number of rows/columns). Land-cover change was then quantified by pixel-wise pairing, whereby each 300 m cell in 1992 was matched to the cell at the same spatial index in 2022, allowing transitions to be computed directly for every pixel across Gabon.

### 2.3. Protected-Area Data

Two datasets were used to map Gabon’s terrestrial protected areas. Official data were retrieved from the World Database on Protected Areas (WDPA), published by the UNEP World Conservation Monitoring Centre (UNEP-WCMC) in June 2025. Community-based conservation areas and Other Effective area-based Conservation Measures (OECMs) were extracted from the file WDPA_WDOECM_Jun2025_Public_GAB_shp-polygons.shp. Only terrestrial polygons were retained for analysis.

### 2.4. Primate Occurrence Data and Processing

Non-human primate occurrence records were compiled from two complementary sources. First, we used field data from CIRMF research missions conducted between 2012 and 2024 (Figure S1 Supplementary Materials) [27,28]. Missions were carried out across all nine provinces of Gabon and yielded 512 georeferenced occurrences. These missions combined targeted reconnaissance surveys and opportunistic sightings, guided by local ecological knowledge (e.g., hunters and experienced field guides). Species identification was primarily based on direct visual observations and, when available, supported by fecal sample collection for molecular confirmation [28], with locations recorded in situ using GPS. Second, we complemented the field dataset with 35 additional validated georeferenced records for Gabon downloaded from the Global Biodiversity Information Facility (GBIF Occurrence Download. Available online: https://doi.org/10.15468/dl.ycyk25; accessed on 13 June 2025), which mainly contributed records for nocturnal taxa (Galagidae and Lorisidae).

All records were harmonized using a common quality-control procedure: we removed duplicate coordinates and excluded records with missing or implausible locations. To limit effort-related sampling bias and spatial autocorrelation arising from clustered detections, we applied spatial thinning by enforcing a minimum nearest-neighbor distance of 1 km between retained occurrences. This step reduces local pseudo-replication in heavily sampled areas and improves the comparability of occurrence-density patterns at the national scale, while approximating a minimum level of independence across taxa with different ranging ecology. After quality control and 1 km thinning, 481 occurrences were retained for subsequent analyses (Table A1, Appendix A).

Because independent ground validation of the 1992 land-cover map is not feasible, we relied on the producer-provided validation of the ESA CCI product and interpreted long-term change primarily through broad, high-magnitude transitions. For the 2022 map, we conducted a field-based plausibility check restricted to the areas visited during CIRMF missions. At surveyed sites, we recorded observable land-cover characteristics (e.g., dominant vegetation structure and canopy cover, presence of cultivation/clearing, built-up features, and water bodies) and compared them with the criteria defined by the FAO Land Cover Classification System (LCCS) used in the Copernicus product. This site-level verification was intended to detect obvious misclassification in visited areas and to confirm the local consistency of the main land-cover classes used in our habitat dynamics analyses.

### 2.5. Land-Cover Change Detection, Transition Matrix Construction, and Statistical Testing of Forest-Loss Drivers

We quantified land-cover composition and change in Gabon using categorical rasters for 1992 and 2022 (CRS: WGS 84; identical extent, resolution, and dimensions), masked to the national boundary. To ensure precise spatial measurements across the national territory, surface areas were calculated by computing per-cell geodesic areas using the *terra::cellSize* function. These individual cell areas were then aggregated by land-cover class through zonal summation. All reported areas and transition statistics in this study are based on these geodesic estimates.

For each year, class-level areas (km^2^) and proportions were derived by dividing class area by the total valid mask area. Absolute and relative changes between 1992 and 2022 were then computed. To assess land-cover transitions, we paired each 1992 pixel with its 2022 class, summing per-cell areas to generate a transition matrix. Row-wise percentages were used to describe how each 1992 class redistributed across 2022 categories.

To assess which land-cover categories were most strongly associated with evergreen forest loss, we analyzed all pixels mapped as evergreen broadleaved forest (class 50) in 1992 that transitioned to a different class by 2022 (*n* = 44,962 pixels). Each forest-loss pixel was assigned to one of three 2022 outcomes: agriculture (30,857 cells), urban (171 cells), or other land-cover types (13,934 cells). We then applied a chi-square goodness-of-fit test comparing the observed distribution of outcomes to expected counts derived from the proportional availability of these categories in the 2022 landscape.

### 2.6. Spatial Analysis of Primate Occurrence Hotspots

To identify areas of high primate occurrence, we applied a two-step spatial analysis. First, we estimated kernel density (KDE) using a fine grid (500 × 500) constrained by the Gabon national boundaries (GADM) and a bandwidth of 0.2° (~22 km). We further verified that the main KDE patterns were stable across alternative bandwidths (0.1–0.3°). This produced a raster of relative occurrence density, from which descriptive statistics and distribution histograms were generated. The KDE allowed us to visualize continuous gradients of primate concentration across the landscape. Second, to statistically validate clusters, we applied the local Getis–Ord Gi* test on a regular grid of 0.05° (~5 km) covering Gabon. Occurrence counts per cell were computed and spatial weights were defined using queen contiguity, where each cell was compared to its eight adjacent neighbors (first-order queen contiguity), corresponding to an approximate maximum centroid-to-centroid distance equal to the cell diagonal (0.05° × √2 ≈ 0.0707°, i.e., ~7–8 km at Gabon’s latitude). Local Gi* z-scores were calculated with *p*-values adjusted by the Benjamini–Hochberg method (FDR). Cells were then classified as hotspots or coldspots at 95% (|z| ≥ 1.96, *p* ≤ 0.05) and 99% confidence (|z| ≥ 2.58, *p* ≤ 0.01), while non-significant areas were treated as background.

### 2.7. Evaluation of the Contribution of Protected Areas to Primate Conservation

We evaluated whether primate occurrences were disproportionately located within Gabon’s protected-area network using a Monte Carlo randomization test. The observed proportion of 481 georeferenced records falling inside protected areas was compared with a null distribution generated under complete spatial randomness (CSR). For each iteration, the same number of points (*n* = 481) was randomly drawn from non-null land-cover cells, and the proportion inside protected areas was calculated. This procedure was repeated 10,000 times, and the *p*-value was defined as the proportion of simulations equal to or exceeding the observed value.

## 3. Results

### 3.1. Diversity and Conservation Status of Fully Protected NHPs in Gabon

A total of ten non-human primate species, fully protected under Gabonese law, are present across the national territory, belonging to the families Hominidae, Cercopithecidae, Galagidae, and Lorisidae (Table 1).

The ten studied species exhibit diverse conservation statuses according to the IUCN Red List. The Hominidae are the most threatened, with *Gorilla gorilla* and *Pan troglodytes* classified as Critically Endangered and Endangered, respectively. Among the Cercopithecidae, *Mandrillus sphinx* is listed as Vulnerable and the endemic *Allochrocebus solatus* as Near Threatened. Within the nocturnal primates (Galagidae and Lorisidae), most taxa are classified as Least Concern, except for *Arctocebus aureus*, which is Near Threatened.

### 3.2. Spatial Distribution and Observation Frequency of Non-Human Primates in Gabon

**Figure 2:**
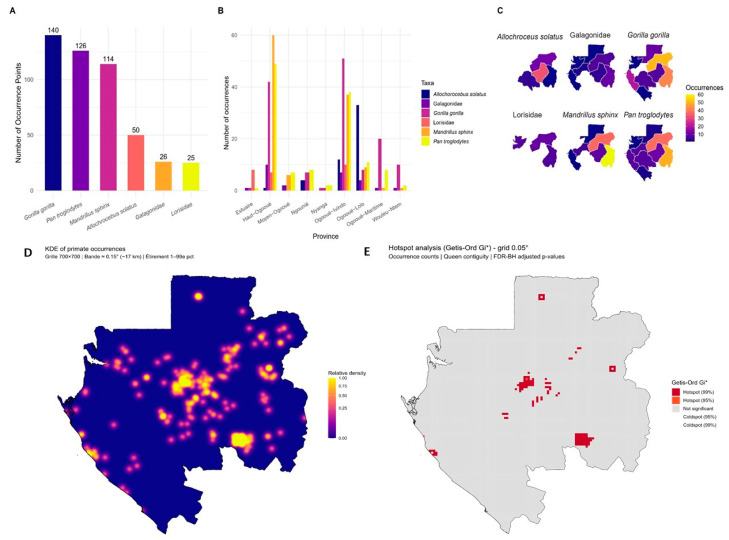
Spatial and taxonomic distribution of primate occurrences in Gabon. (**A**) Total number of primate occurrences by species; (**B**) number of occurrences by species and province; (**C**) distribution of occurrences by province and species; (**D**) primate density; (**E**) primate hotspots.

A total of 481 georeferenced occurrence records were compiled across Gabon (Figure 2A). Three species account for nearly 80% of observations: *Gorilla gorilla* (29.1%), *Pan troglodytes* (26.2%), and *Mandrillus sphinx* (23.7%). Other taxa were less represented, including *Allochrocebus solatus* (10.4%), Galagidae (5.4%), and Lorisidae (5.2%). Spatial analysis by province revealed pronounced regional heterogeneity (Figure 2B,C). Ogooué-Ivindo emerged as a major concentration area, with high counts for *G. gorilla* (*n* = 51), *P. troglodytes* (*n* = 38), *M. sphinx* (*n* = 37), and *A. solatus* (*n* = 12), alongside notable records of Galagidae (*n* = 7) and Lorisidae (*n* = 10). Haut-Ogooué also exhibited elevated totals dominated by *M. sphinx* (*n* = 60), followed by *P. troglodytes* (*n* = 49), *G. gorilla* (*n* = 42), Galagidae (*n* = 10), and Lorisidae (*n* = 7). Other provinces, such as Ogooué-Lolo and Ogooué-Maritime, presented moderate but compositionally diverse assemblages, whereas Nyanga and Ngounié yielded only sporadic records, suggesting restricted diversity and/or under-sampling.

Kernel density estimation (KDE) refined this provincial overview by highlighting concentrated clusters of primate occurrences. Approximately 10% of Gabon’s surface (≈ 26,700 km^2^) was located above the 90th percentile of density values and 5% (≈ 13,300 km^2^) above the 95th percentile, with the largest continuous hotspot exceeding 13,000 km^2^ (Figure 2D). Complementary hotspot detection using the Getis–Ord Gi* statistic confirmed the presence of statistically significant clusters: although most grid cells were not significant (98.5%), 1.5% were identified as hotspots at the 99% confidence level and 0.02% at the 95% level (Figure 2E).

### 3.3. Primate Occurrences Across Gabon’s Land-Cover Classes

**Figure 3:**
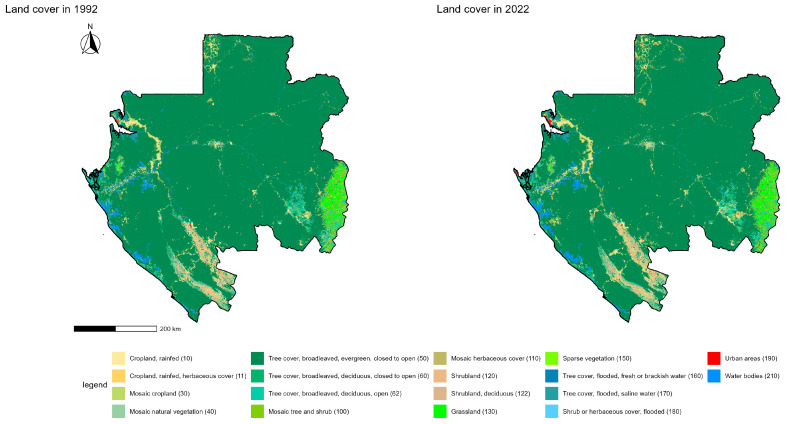
Land-cover mapping.

At the national scale, Gabon’s land cover (1992–2022), harmonized to the FAO Land Cover Classification System (LCCS), spans a broad range of vegetation types, croplands, built-up areas, and aquatic systems (Figure 3; Table 2). All classes mapped in 1992 were still represented in 2022 except sparse vegetation (class 150), which was no longer mapped at a 300 m resolution in the 2022 product.

The analysis of the primate occurrences revealed a strong dependence on evergreen broadleaved forest (class 50), which concentrated 423 points, representing 87.9% of all records. Other land-cover classes were only marginally represented: 3.1% of occurrences were located in open deciduous forest (class 62), 1.9% in mosaic natural vegetation (class 40), 1.7% in rainfed croplands (class 10), and 1.5% in herbaceous croplands (class 11). A few isolated occurrences (<1%) were detected in atypical environments, including water bodies (class 210), mosaic croplands (class 30), saline flooded forest (class 170), and urban areas (class 190), as well as sporadically in mosaic herbaceous (class 110) and flooded shrub/herbaceous formations (class 180).

### 3.4. Dynamics of the Principal Primate Habitat (Evergreen Broadleaved Forest, Class 50)

**Figure 4:**
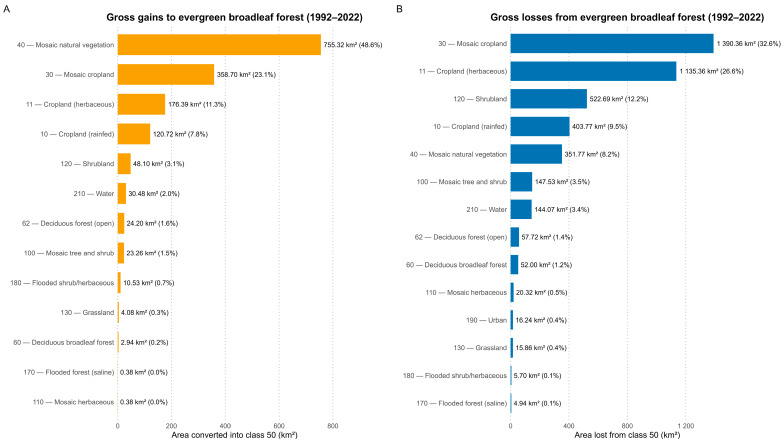
Transition matrix of evergreen broadleaved forest (class 50) between 1992 and 2022. (**A**) gross gains to evergreen to evergreen broadleaf forest; (**B**) Gross losses from evergreen broadleaf forest.

The transition analysis shows a very high persistence of evergreen broadleaved forest (class 50) between 1992 and 2022: 50 to 50 = 223,476 km^2^ (98.13% of its 1992 extent, 227,744.2 km^2^). Gross losses from class 50 totaled 4046.58 km^2^ (1.87%), dominated by conversions to cropland classes 10, 11, 30 combined = 2777.13 km^2^ (68.63% of gross losses) followed by shrubland (120: 495.72 km^2^; 12.25%), mosaic natural vegetation (40: 333.45 km^2^; 8.24%), mosaic tree and shrub (100: 139.86 km^2^; 3.46%), and water (210: 136.62 km^2^; 3.38%). Minor pathways included shifts to deciduous/open broadleaf forests (60 + 62: 104.04 km^2^; 2.57%), mosaic herbaceous (110: 19.26 km^2^; 0.48%), urban (190: 15.39 km^2^; 0.38%), grassland (130: 15.03 km^2^; 0.37%), and flooded classes (170 + 180: 10.08 km^2^; 0.25%) (Figure 4A).

Gains into class 50 summed to 1474.92 km^2^ (0.68%), driven mainly by mosaic natural vegetation (40: 716.22 km^2^; 48.56% of gains), mosaic cropland (30: 340.11 km^2^; 23.06%), and rainfed croplands (11: 167.22 km^2^; 10 and 10: 114.48 km^2^; 19.10%), combined with smaller inputs from 120 (45.63 km^2^), 62 (22.95 km^2^), 100 (22.05 km^2^), and 210 (28.89 km^2^). The net balance for class 50 over 1992–2022 was −2571.66 km^2^ (≈ −1.19% relative to 1992; −1.022 percentage points of national area), indicating a modest contraction while remaining the dominant national habitat (Figure 4B).

Evergreen broadleaved forest loss was overwhelmingly associated with conversion to agriculture, which accounted for 30,857 of the 44,962 forest-loss pixels, compared with 13,934 transitioning to other land-cover types and only 171 to urban areas. This distribution differed strongly from the expected frequencies under the null model (χ^2^ = 31,525; df = 2; *p* < 0.001), indicating that forest attrition during 1992–2022 was disproportionately linked to agricultural expansion. Standardized residuals confirmed agricultural transitions as over-represented relative to expectation, whereas urban and “other” outcomes were under-represented.

### 3.5. Land-Cover Stability and Transitions Around Primate Occurrences (1992–2022)

**Figure 5:**
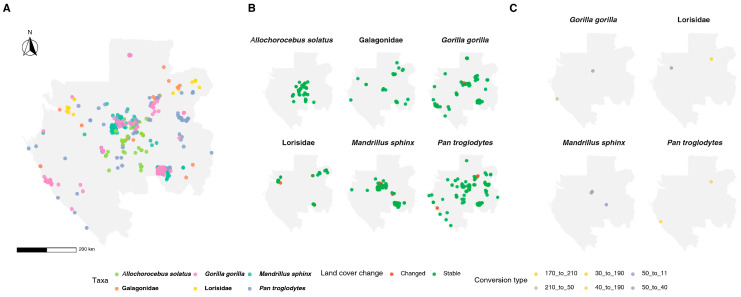
Spatial patterns of land-cover and primate occurrences in Gabon. (**A**) Overall spatial distribution of primate occurrences; (**B**) localized land-cover changes at primate occurrence points; (**C**) land-cover conversions at primate occurrence points.

More than 90% of primate records were associated with locations where land cover remained unchanged between 1992 and 2022 (Figure 5). Stable sites were overwhelmingly evergreen broadleaved forest (class 50), which hosted occurrences of all taxa, including the highest frequencies for *Gorilla gorilla*, *Pan troglodytes*, and *Mandrillus sphinx*.

In contrast, only a small fraction of records fell in cells that changed class over the period, involving rare transitions from evergreen forest to mosaic natural vegetation or rainfed croplands, isolated urban conversions, and occasional swaps among flooded and open-water classes.

### 3.6. Effectiveness of the Protected-Area Network for Primate Conservation

**Figure 6:**
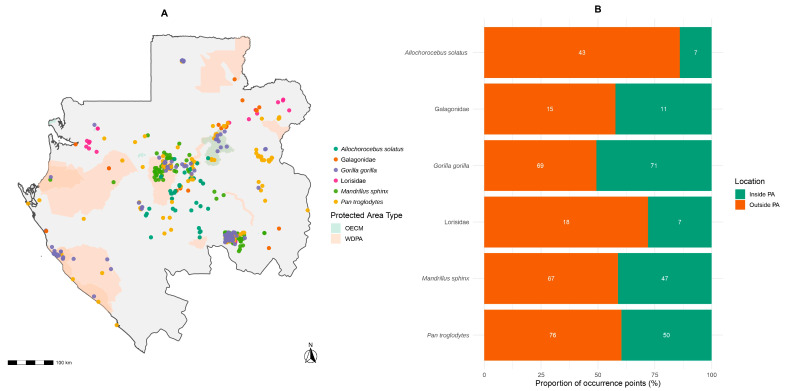
Protected areas and non-human primate distribution in Gabon. (**A**) Spatial distribution of non-human primate occurrences overlaid on protected areas and (**B**) number of primate species represented by protection status (official, non-official, or unprotected).

Of the 481 primate records compiled across Gabon, 193 (40.1%) occurred inside terrestrial protected areas (WDPA + OECM), versus 288 (59.9%) outside (Figure 6). Species-specific patterns varied; *Gorilla gorilla* showed 50.7% of records inside PAs, *Pan troglodytes* 39.7%, and *Mandrillus sphinx* 41.2%, whereas the endemic *Allochrocebus solatus* was predominantly outside (86%). Nocturnal taxa were similarly underrepresented in PAs, with 42.3% of Galagidae and 28% of Lorisidae records inside (Figure 6).

Monte Carlo randomization under complete spatial randomness (CSR; terrestrial mask) indicated that only 18.47% of points would be expected inside PAs by chance (≈89/481). The observed 40.1% corresponds to an enrichment ≈ 2.17× (*p* < 0.001), demonstrating that primate occurrences fall within protected areas more than twice as often as predicted by the null model.

## 4. Discussion

Gabon stands out as a major stronghold for primate conservation. It supports a substantial share of the global population of both the Central African chimpanzee (*Pan troglodytes troglodytes*) and the Western lowland gorilla (*Gorilla gorilla gorilla*), while also hosting some of the largest known mandrill aggregations [14,36,37]. These unique biological assets place a critical international responsibility on Gabon for the long-term survival of these threatened taxa. This responsibility is further reinforced by the endemism and restricted range of the sun-tailed monkey (*Allochrocebus solatus*) in central Gabon, which increases its susceptibility to localized disturbance [38]. In this context, the two hominids (*Gorilla gorilla* and *Pan troglodytes*) represent the highest conservation priorities. Our results reveal that nearly 50% of their occurrences are located outside protected areas, a particularly worrying finding given their status as threatened species according to the IUCN Red List. This exposure is critical considering the well-documented pressures on great apes in equatorial Africa, including direct persecution, the expansion of the human footprint, and diseases, all of which are aggravated by their slow life-history traits that limit population recovery [39]. Within Cercopithecidae, the contrast between *Mandrillus sphinx* (Vulnerable) and *A. solatus* (Near Threatened) further indicates that conservation priorities should not be limited to great apes alone but should also account for endemism and restricted distributions. Finally, although most nocturnal primates (Galagidae and Lorisidae) are currently classified as Least Concern, this should not be equated with low conservation relevance, as these taxa remain comparatively under-studied, and population trends may be poorly documented. This uncertainty is exemplified by Allen’s galago (*Sciurocheirus alleni*), which is listed in national biodiversity records for Gabon [40], whereas recent IUCN distribution syntheses focus on southeastern Nigeria, Cameroon, and Bioko without explicitly including Gabon [41].

Our nationwide analysis confirms that Gabon still retains a large core of suitable habitats for non-human primates: evergreen broadleaved forest (class 50), the principal primate habitat in the country, exhibited very high persistence (98.13%) over 1992–2022. In parallel, the spatial analysis highlights extensive concentrations in Ogooué-Ivindo and Haut-Ogooué, which emerge as priority landscapes. This result aligns with previous studies showing high species richness and a non-uniform distribution of non-human primates across the Gabonese territory [10,11,12,28,42].

The pre-eminence of agricultural conversion that we observe accounting for 68.63% of gross forest loss aligns with Congo Basin syntheses showing that small-scale, non-mechanized agriculture accounts for most recent forest disturbance [43,44]. Furthermore, transport infrastructure amplifies these pressures by increasing access, generating edge effects, and fragmenting habitat [45]. Substantial literature shows that the majority of forest loss concentrates near transport corridors and along expanding networks, including logging roads [46]. It is therefore crucial to anticipate interface zones where projects under the National Development Plan for the Transition (PNDT), including road and rail developments, intersect high-value primate habitats. In this context, developing a decadal (or annual) land-cover time series would be a valuable extension for future work, enabling reconstruction of the timing of major transitions and a more explicit assessment of potential correlations between land-cover dynamics and observed occurrence patterns. Such a time series would also help distinguish recent, rapid conversions from slower cumulative change, thereby improving risk forecasting for primate habitats under planned infrastructure expansion.

Connectivity is the other critical dimension: connected ecological networks support gene flow, recolonization, and long-term demographic viability. IUCN connectivity guidelines explicitly recommend recognizing and safeguarding ecological corridors to complement protected areas and OECMs, an approach directly relevant to the clusters we mapped and to future development corridors in Gabon [47].

Our Monte Carlo enrichment (≈2.17×; *p* < 0.001) shows that primate records occur more than twice as often inside protected areas than expected under CSR (18.47% expected vs. 40.1% observed), indicating that Gabon’s PA/OECM network makes a concrete contribution to retaining a primate presence. This is consistent with global assessments showing that, despite variation across contexts and management, protected areas reduce biodiversity loss relative to unprotected lands [48,49,50]. Nevertheless, species-level gaps remain, for example, *Allochorocebus solatus*, with 86% of records outside PAs pointing to representativeness shortfalls that corridor planning and targeted landscape tools could address.

Beyond classical PAs, Other Effective area-based Conservation Measure (OECM) areas managed to deliver sustained positive outcomes for biodiversity even if not primarily for conservation offer a recognized pathway to secure complementary habitat [51,52]. Maintaining up-to-date WDPA/WD-OECM baselines is therefore essential as PNDT projects roll out and spatial safeguards are refined (Protected Planet WDPA/OECM) [53,54].

Three caveats frame interpretation. (1) Sampling bias (accessibility and proximity to parks/research sites) can inflate densities and clusters. (2) Our CSR null is conservative; inhomogeneous nulls (constrained to persistent forest or weighted by accessibility) would sharpen inference about PA capture without changing the headline result.

Three priorities follow directly from our analysis: (i) safeguard and formalize connectivity between “hotspot” landscapes through ecological corridors embedded in national and provincial land-use plans, consistent with IUCN guidance and WDPA/OECM standards; (ii) steer PNDT investments in transport and productive sectors away from high-value habitats (avoidance first) and, where linear infrastructure is unavoidable, deploy impact-minimizing designs (alignment optimization, roadless buffers, wildlife crossings, and access management) grounded in the well-documented roads deforestation link; and (iii) target out-of-PA gaps for species such as *Allochorocebus solatus* via a mix of new or expanded protected areas, community- or privately governed OECMs, and corridor agreements, recognizing that area quality and placement matter at least as much as nominal coverage for securing conservation outcomes [55,56].

## 5. Conclusions

In summary, Gabon serves as a vital sanctuary for ten primate species granted full legal protection, the most vulnerable of which are the Western lowland gorilla and the Central chimpanzee. Their primary habitat, the evergreen forest, remained largely stable between 1992 and 2022, although agricultural expansion was identified as the main driver of localized forest loss. Our mapping highlights critical concentration hotspots in the Ogooué-Ivindo and Haut-Ogooué Provinces and demonstrates that the national protected-area network fulfills its role effectively, capturing a significantly higher proportion of species occurrences than would be expected by chance. In practice, territorial planning should embed connectivity by securing corridors between density cores, avoid placing PNDT infrastructure in or adjacent to hotspots, and where avoidance is impossible minimize impacts through alignment optimization, wildlife crossings, and access management. Complementary, well-sited OECMs and, where needed, boundary adjustments to protected areas can close taxonomic and functional gaps. Looking ahead, future work will develop species distribution models (SDMs) corrected for sampling bias, evaluated in ensemble frameworks and coupled with land-use and climate scenarios, then linked to connectivity analyses to prioritize corridors, guide restoration, and optimize OECM siting or expansion of the protected-area network.

## Figures and Tables

**Table 1:** List of primate species fully protected in Gabon and their conservation status according to the IUCN.

Common Name	Scientific Name	Family	IUCN Status	References
Chimpanzee	*Pan troglodytes*	Hominidae	Endangered (EN)	[13]
Western Gorilla	*Gorilla gorilla*	Hominidae	Critically Endangered (CR)	[12]
Mandrill	*Mandrillus sphinx*	Cercopithecidae	Vulnerable (VU)	[14]
Sun-tailed monkey	*Allochrocebus solatus*	Cercopithecidae	Near Threatened (NT)	[29]
Elegant Galago	*Euoticus elegantulus*	Galagidae	Least Concern (LC)	[30]
Demidoff’s Galago	*Galagoides demidoff*	Galagidae	Least Concern (LC)	[31]
Gabon Squirrel Galago	*Sciurocheirus gabonensis*	Galagidae	Least Concern (LC)	[32]
Thomas’s Dwarf Galago	*Galagoides thomasi*	Galagidae	Least Concern (LC)	[33]
Golden Angwantibo	*Arctocebus aureus*	Lorisidae	Near Threatened (NT)	[34]
Milne-Edwards’s Potto	*Perodicticus edwardsi*	Lorisidae	Least Concern (LC)	[35]

**Table 2:** Quantitative changes in land-cover classes in Gabon between 1992 and 2022.

Class	Class Description	Area 1992 (km^2^)	Proportion 1992 (%)	Area 2022 (km^2^)	Proportion 2022 (%)	Change in Area (km^2^)	Change in Proportion (%)
10	Cropland, rainfed	2904.8	1.095	3078.9	1.160	174.1	0.066
11	Cropland, rainfed, herbaceous cover	2536	0.956	3472.9	1.309	936.9	0.353
30	Mosaic cropland	4161.3	1.568	5235.6	1.973	1074.3	0.405
40	Mosaic natural vegetation	2247.5	0.847	1703.8	0.642	−543.7	−0.205
50	Tree, broadleaved, evergreen, closed to open	227,744.2	85.829	225,031.3	84.807	−2712.8	−1.022
60	Tree, broadleaved, deciduous, closed to open	115.5	0.044	750.5	0.283	635	0.239
62	Tree, broadleaved, deciduous, open	6798	2.562	6503.2	2.451	−294.8	−0.111
100	Mosaic tree and shrub	2880.9	1.086	3202.8	1.207	321.9	0.121
110	Mosaic herbaceous	927.9	0.350	817.9	0.308	−110	−0.041
120	Shrubland	4687.7	1.767	5012.7	1.889	325	0.122
122	Shrubland, deciduous	0.3	0.000	0.1	0.000	−0.2	0
130	Grassland	3761	1.417	3691.3	1.391	−69.7	−0.026
150	Sparse vegetation	0.6	0.000	—	—		
160	Tree cover, flooded, fresh or brackish water	310.2	0.117	308.2	0.116	−2	−0.001
170	Tree cover, flooded, saline water	2619.1	0.987	2493.4	0.940	−125.7	−0.047
180	Shrub or herbaceous cover, flooded	294.2	0.111	201.2	0.076	−93.1	−0.035
190	Urban	96.3	0.036	273.5	0.103	177.2	0.067
210	Water	3259.4	1.228	3567.5	1.344	308.1	0.116

This table summarizes the changes in surface area and proportional extent of land-cover classes between 1992 and 2022, based on data from the ESA Climate Change Initiative (CCI) and Copernicus (spatial resolution: 300 m). Classes are coded according to the FAO Land Cover Classification System (LCCS). Class = numerical code of the land-cover class; class description = designation of the land-cover type; area 1992 (km^2^) = total area in 1992; proportion 1992 (%) = relative share in 1992; area 2022 (km^2^) = total area in 2022; proportion 2022 (%) = relative share in 2022; change in area (km^2^) = absolute change in surface area; change in proportion (%) = relative change in proportion between 1992 and 2022.

## Data Availability

The original contributions presented in the study are included in the article. Further inquiries can be directed to the corresponding author. The scripts used for statistical analysis and supplementary occurrence data are available from the corresponding author upon reasonable request.

## Supplementary Materials

The following supporting information can be downloaded at: https://www.mdpi.com/article/10.3390/biology15050405/s1, Figure S1: Survey coverage prox.

## Abbreviations

NHP	Non-Human Primates
LUC	Land Use/Cover (Land Use and Land Cover)
LCCS	Land Cover Classification System (FAO)
ESA	European Space Agency
ESA CCI	European Space Agency Climate Change Initiative
C3S	Copernicus Climate Change Service
WDPA	World Database on Protected Areas
UNEP-WCMC	United Nations Environment Programme—World Conservation Monitoring Centre
OECM	Other Effective area-based Conservation Measures
GBIF	Global Biodiversity Information Facility
CIRMF	Centre Interdisciplinaire de Recherches Médicales de Franceville
GADM	Global Administrative Areas (database)
KDE	Kernel Density Estimation
Gi*	Getis–Ord Gi* (local hotspot statistic)
FDR	False Discovery Rate (Benjamini–Hochberg adjustment)
CSR	Complete Spatial Randomness
IUCN	International Union for Conservation of Nature
PNDT	National Development Plan for the Transition (Plan National de Développement pour la Transition)
GPS	Global Positioning System
CRS	Coordinate Reference System

## Appendix A

**Table A1:** Source-specific contribution of occurrence records by taxon (CIRMF vs. GBIF).

Taxon	Total	CIRMF (2012–2023)	GBIF
*Gorilla gorilla*	140	140	0
*Pan troglodytes*	126	126	0
*Mandrillus sphinx*	114	114	0
*Allochrocebus solatus*	50	50	0
Galagidae	26	13	13
Lorisidae	25	8	17
Total	481	451	30
