# Supplementary material for: Non-Human Primates in Gabon: Occurrence Hotspots, Habitat Dynamics, Protected-Area Performance, and Conservation Challenges
Source: Biology (Basel). 2026 Feb 28;15(5):405. doi: 10.3390/biology15050405 (PMC12985318; doi:10.3390/biology15050405)
Supplement: Supplementary file 1 [file biology-15-00405-s001.zip › biology-4131086-supplementary.pdf]

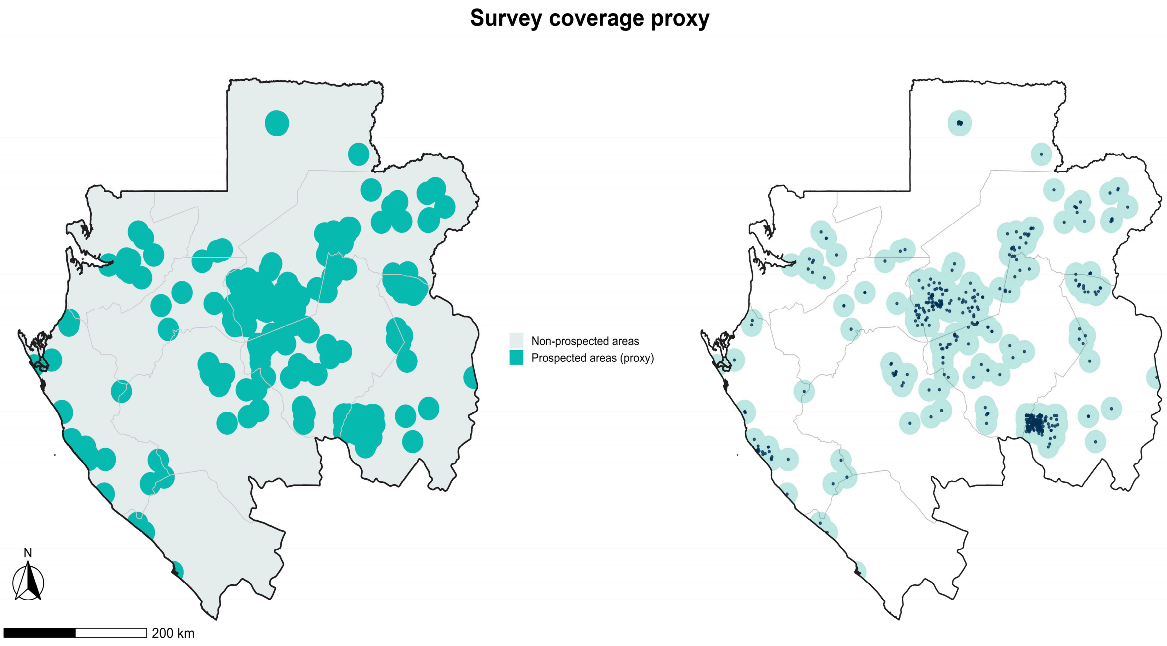

Figure S1. Survey coverage proxy. Prospected areas (green) represent the spatial footprint of field prospection, Grey areas indicate regions that were not prospected during the study period.
